# Responses of weed community, soil nutrients, and microbes to different weed management practices in a fallow field in Northern China

**DOI:** 10.7717/peerj.7650

**Published:** 2019-09-06

**Authors:** Xian Gu, Yu Cen, Liyue Guo, Caihong Li, Han Yuan, Ziwen Xu, Gaoming Jiang

**Affiliations:** 1 State Key Laboratory of Vegetation and Environment Change, Institute of Botany, the Chinese Academy of Sciences, Beijing, China; 2 College of Resources and Environment, University of Chinese Academy of Sciences, Beijing, China

**Keywords:** Fallow field, Weed seed bank, Soil microorganisms, Glyphosate, Organic farming

## Abstract

The long-term use of herbicides to remove weeds in fallow croplands can impair soil biodiversity, affect the quality of agricultural products, and threaten human health. Consequently, the identification of methods that can effectively limit the weed seed bank and maintain fallow soil fertility without causing soil pollution for the next planting is a critical task. In this study, four weeding treatments were established based on different degrees of disturbance to the topsoil: natural fallow (N), physical clearance (C), deep tillage (D), and sprayed herbicide (H). The changes in the soil weed seed banks, soil nutrients, and soil microbial biomass were carefully investigated. During the fallow period, the C treatment decreased the annual and biennial weed seed bank by 34% against pretreatment, whereas the H treatment did not effectively reduce the weed seed bank. The D treatment had positive effects on the soil fertility, increasing the available nitrogen 108% over that found in the N soil. In addition, a pre-winter deep tillage interfered with the rhizome propagation of perennial weeds. The total biomass of soil bacterial, fungal, and actinomycete in H treatment was the lowest among the four treatments. The biomass of arbuscular mycorrhizal fungi in the N treatment was respectively 42%, 35%, and 91%, higher than that in the C, D, and H treatments. An ecological weeding strategy was proposed based on our findings, which called for exhausting seed banks, blocking seed transmission, and taking advantage of natural opportunities to prevent weed growth for fallow lands. This study could provide a theoretical basis for weed management in fallow fields and organic farming systems.

## Introduction

Fallow tillage is an ancient agronomic practice that has been reintroduced for the restoration of arable lands and to solve the problems of cropland degradation, groundwater depletion, and environmental pollution caused by conventional agricultural practices (Manalil & Flower, 2014; Ministry of Agriculture of the People’s Republic of China, 2016; Pinto, Fernández Long & Piñeiro, 2017). However, weeds become a problem in a fallow cropland with their ability to adapt and evolve rapidly, causing enormous economic losses to agriculture annually. For these reasons, weeds have been referred to as the “Red Queen” in farmlands (Vigueira, Olsen & Caicedo, 2013). However, weeds are essential elements of agroecosystem that support biodiversity within a crop field, provide shelter to predators of pests, and improve soil fertility with the input of dead roots or litter (Marshall et al., 2003). Moreover, a variety of weeds can restrict the invasion of a single weed. Suitable weed cover can also reduce soil erosion and improve soil chemical properties (Wortman et al., 2010).

Chemical weeding of fallow fields can be effective in the short term, however, the doses of herbicides have increased with their long-term use. Weed biodiversity is also reduced, leading rare species to disappear (Gaba et al., 2016). In China, 27 herbicide-resistant field weeds have been identified, including *Descurainia sophia*, *Conyza canadensis*, and *Digitaria sanguinalis* (http://www.weedscience.org/). Glyphosate, paraquat, and other sterilant herbicides are widely used for weed control but cause serious environmental pollution and human health problems. These issues are compounded by the misuse and abuse of herbicides that is found among farmers (Zhang, 2013). Glyphosate, with a half-life that ranges from a few days to 2 or 3 months, can limit soil enzyme activity and alter microbial communities (De Andréa et al., 2003; Lancaster et al., 2010), leading to declines in land quality and food safety. Globally, 41 species of weeds have developed resistance to glyphosate. For example, *Amaranthus palmeri* can reach a height of 2.5 m with a daily average growth of more than six cm in America, despite being treated by glyphosate (A growing problem, 2014). Therefore, the evolution of weed resistance that is induced by herbicide use poses a number of challenges to chemical control. For instance, 258 total weed species (150 dicotyledonous and 108 monocotyledonous weeds) have evolved an herbicide resistance worldwide, primarily due to the abuse of herbicides (http://www.weedscience.org/). Moreover, glyphosate is classified as a Category 2A carcinogen (Guyton et al., 2015) and can cause nonalcoholic fatty liver (Mesnage et al., 2017).

A fallow strategy is more widely used in organic farming than in a conventional agriculture. During the 2–3 year conversion to organic farming, fallowing is generally applied to improve soil conditions and eliminate harmful substances. However, the development of organic agriculture is held up by the inability to determine the balance in the optimal control of weeds to benefit the agroecosystem. The weeds in a fallowed land are usually managed either by herbicides or tillage (Manalil & Flower, 2014). Weed management in organic agriculture requires the full application of ecological principles, which includes the development of nonchemical approaches to limit weed density while meeting an economic threshold. The soil weed seed bank is the primary source for a weed explosion on farmland (Qiang, 2009), making it the primary target of weed management in the future. Tillage at different intensities may affect annual or perennial emergence of weeds (Gruber & Claupein, 2009). For example, conservation tillage can lead to increases in weed seed density and diversity, whereas mild disturbances cause dormancy in the weed seed bank (Santín-Montanyá et al., 2016).

Actually, weeds under a fallow condition play an important role in the agroecosystem. It was reported that, fallowing with weeds reduces nitrogen losses and increases soil organic matter, compared to continuous cropping (Ando et al., 2014; Wortman, 2016). The concept of using weeds to inhibit weeds has been tested and includes planting leguminous herbs to control other weeds and increase crop yields (Hauser, Nolte & Carsky, 2006; Promsakha Na Sakonnakhon et al., 2006). However, few reports have examined measures that both prevent and control fallow weeds. A rotational fallow can inhibit the growth of perennial weeds, while the density of annual weeds, such as *Chenopodium album*, may increase (Hume, 1982). Some found that there was no difference in the composition of the weed community by different tillage practices in the fallow lands (Derksen et al., 1994). The exploration in the effects of herbicides on fallow fields, the reduction of weed seed banks, and the use of environmentally healthy alternatives to herbicides must be explored in order to stop the normalization of herbicide abuse.

The amount of weeds in fallow lands far exceed those of nonfallow lands, which is a problem compounded by the long-term dormancy of some weed seeds. Chemical weeding may limit the degree of a weed explosion but causes serious environmental pollution. It is important to control the damage done by both weeds and herbicides in the development of organic agriculture by reducing the capacity of weed seed banks. We hypothesized that repeated soil disturbances with physical methods could prevent the normal succession of weed communities by cutting the rhizomes or killing the seedlings of weeds at the earliest time as well as consuming the weed seed bank, thus stopping the development of weeds and their seed production. Physical methods could also benefit soil nutrients and the microbial diversity of the soil, while chemical practices do not. The aim of this investigation was to identify the most suitable approach to reducing weed seed banks with limited herbicide pollution. This study may provide ecological solutions for weed management in both fallowing and organic farming practices.

## Materials and Methods

### Study site

The field experiment was conducted in Meng Village, Daliang Town, Wuqing District, Tianjin City in Northern China (39°34′9.94″N, 116°59′49.65″E), and was approved by the Plant Eco-physiological Research Group in the Institute of Botany, Chinese Academy of Sciences (Project Number: 201601.8). The village is in a lower reach of an alluvial plain with an elevation of 2.8–13 m. The area has a temperate semihumid continental monsoon climate with the annual average temperature of 12.2 °C, an annual precipitation of 557.3 mm, and a frost-free period of 212 days (Wuqing District People’s Government (WDPG), 2019). The soil acidity is somewhat neutral (pH ~7.5). Wuqing District is one of 800 major grain-producing counties in China, with a farming system of summer maize-winter wheat rotation. In 2011, the local government changed the cropland in the study area to Chinese herbal medicine, resulting in an overgrowth of weeds throughout in the majority of the landscape due to poor management. In 2015, the land was transferred to an ecofarm and replanted with crops, vegetables, and fruit trees. However, after a harvest of radishes (*Raphanus sativus*) in November 2015, no further crops were planted.

### Experimental design

The experiment was conducted from July 2016 to April 2017, with all treatments being arranged in a randomized complete block design with three replicates (plot size, 2.5 × 4 m). Each plot had a two m wide protective belt. The four treatments were as follows: natural fallow, naturally vegetated without any intervention (N); physical clearance, clearing weeds with a hoe and only disturbing the soil surface (C); deep tillage, tillage of the topsoil layer to 20–30 cm with a spade (D); sprayed herbicide, direct spray with glyphosate ammonium salt herbicide (30%) (H). Glyphosate is an herbicide that destroys plants based on internal absorption conduction. The spray concentration of two ml l^−1^, was selected based on the dosage used by local farmers. The herbicide was applied on July 8, August 24, and October 28, 2016, and on April 15, 2017.

Herbicides and a light physical clearing of the soil could not thoroughly eliminate the perennial weeds due to their underground rhizomes. To study the effect of large-area deep plowing on perennial weeds (i.e., *Calystegia hederacea*), deep tillage with a moldboard plow (D) was applied to another area (100 m^2^) of fallow land on the farm in late November 2016. The same plot size with natural fallow (N) was also examined for contrast. *Calystegia hederacea*, a malignant perennial weed in the local farmlands, has horizontal underground rhizomes. The rhizomes with nodes quickly reproduce new shoots, growing easily in sandy soil. In the following spring (early April 2017), the rhizome morphology in the 0–20 cm soil layer was investigated for both treatments. Each treatment included six samples (sample size 0.5 × 0.5 m). Ten rhizome segments were randomly examined in each sample. The diameter and length of each bud in the rhizomes were measured by vernier caliper.

### Weed community and seed bank survey

Weed communities were investigated on July 8, August 24, and October 28, 2016, and on April 15, 2017, before using herbicides. Weed species and plant numbers were recorded for each treatment in three replicates. Weed biomass was determined after drying for 48 h at 65 °C.

Soil seed banks relied on greenhouse germination methods (Gross, 1990). Three soil cores were collected from the soil layer (0–20 cm) with a drill (inside diameter = 2 cm) from each plot before the start of the trial in July 2016 and after the trial in April 2017. As the weed seed bank in fallow land was very rich and uneven, in order to reduce artificial sampling errors and thoroughly stimulated the various weed seeds germination, soil samples in each treatment for this special purpose were pooled and evenly divided into nine portions. The plant residues and small stones were removed and the remaining soil was placed in a greenhouse (18/8 °C, day/night) for seed germination. When there was no weed seedlings on the trays, we placed the soils in a 4 °C cold room for 3 weeks followed by 1 week at alternating temperatures (15 and 4 °C, day and night) (Cardina, Herms & Doohan, 2002) and the soils were stirred in order to continue germination for the remaining seeds. This process was repeated until no additional seedlings emerged. The total number of weed seeding was counted every time.

### Soil nutrients and microorganisms

The weed species in the experiment field are most abundant in October of each year. To investigate the effect of weed communities on soil properties, we collected three soil samples from each plot on October 15, 2016. A portion of each fresh soil sample (0–20 cm) was reserved for soil microbial determination. Soil total nitrogen was determined by the digestion–distillation method (Bremner & Tabatabai, 1972) through FOSS KJELTEC 8200 (Foss, Hilleroed, Denmark). The total phosphorus was measured by spectrophotometer after sulfuric–perchloric acid digestion (Sommers & Nelson, 1972). The standard reference materials (SRM) for soil total nitrogen and soil total phosphorus were adopted by the GSS-13 (General Administration of Quality Supervision, Inspection and Quarantine of the People’s Republic of China, 1996a) and the SRM of testing recovery was 95% and 102%. A total of 10 g fresh soil samples without roots were soaked with 50 ml KCl of two mol l^−1^ and shook for 30 min, with ammonium nitrogen and nitrate nitrogen being measured by leaching-continuous flow analyzer (Best, 1976; Crooke & Simpson, 1971). The SRM of testing recovery was 101%. Available phosphorus in soil samples was extracted with 0.5M NaHCO_3_ and determined colorimetrically using the molybdate method (Olsen, 1954). The SRM of available phosphorus was adopted by the ASA-2a (General Administration of Quality Supervision, Inspection and Quarantine of the People’s Republic of China, 1996b) and the testing recovery was 97%.

Soil microbial biomass was extracted from the freeze dried soil samples at −80 °C before processing. Total lipids were extracted from moist soil using a chloroform–methanol approach and microbial phosphor lipid fatty acid (PLFA) was measured using a single-phase mixture of chloroform–methanol–citrate buffer (1:2:0.8, v/v/v). PLFA was then purified from the lipid extracts, quantified, and identified (Bossio et al., 1998). Nonadecanoic acid methyl ester (19:0) was used as the internal standard for an Agilent 6850 Gas Chromatograph with the Sherlock Microbial Identification System V4.5 (MIDI, Inc., Newark, DE, USA). The primary microbes were classified into seven groups (Bossio et al., 1998; Fierer, Schimel & Holden, 2003; Frostegård & Bååth, 1996): most common bacteria: 14:0, 15:0, 16:0, 17:0, 18:0; gram-negative: cy17:0, cy19:0, 15:1w4c, 15:1w6c, 16:1w9c, 18:1w7c, 18:1w9c; gram-positive: i14:0, i15:0, a15:0, i16:0, i17:0, a17:0; fungi: 18:2w6c, 18:2w9c, 18:3w3c, 18:3w6c; arbuscular mycorrhizae: 16:1w5c (Olsson, 1999); actinomycetes: 10Me16:0, 10Me17:0, 10Me18:0; protozoa: 20:2w6, 9c, 20:3w6, 9, 12c, 20:4w6, 9, 12, 15c.

## Statistical analyses

The relative importance values (RIV, %) of weeds were computed using the following equation (Caamal-Maldonado et al., 2001):

$${\rm{RIV}} = {{{\rm{Relative\, biomass}} + {\rm{Relative\, frequency}} + {\rm{Relative\, abundances}}} \over 3} \times 100$$

where relative biomass (%) is the ratio of biomass of one species to the biomass of the total; relative frequency (%) is the ratio of the frequency of one species to the frequency of the total; Relative abundance (%) is the ratio of the number of individuals of one species to the total individuals of all species.

The statistical analysis was performed using the SPSS 20.0 statistical software package (IBM Corp, Armonk, NY, USA). A paired-sample *t*-test was performed on the shape of the rhizomes of *Calystegia hederacea*. The weed seed bank, soil nutrients, and soil microbial data were analyzed by one-way ANOVA. A least significance difference test was used to determine the significant differences (*P* < 0.05 and *P* < 0.01). The relationship between the RIV of specific grasses and soil properties was calculated by Pearson correlation analysis. PLFAs observed in each sample were recognized with principal components analysis (PCA) by the correlation matrix (Past Version 3.25, Hammer; University of Oslo, Oslo, Norway).

## Results

### Weed community

A total of 27 weed species were found in the experimental field, including 17 annual, three biennial, and seven perennial weeds (Table 1). The natural fallow treatment (N) contained 18 weed species, which was the lowest number among the treatments. A total of 19 species were noted in the herbicide treatment (H), while 21 and 23 weed species appeared in the physical clearance (C) and the deep tillage (D) treatments, respectively.

**Table 1:** Relative importance values (%RIV) for all types of weeds under the different weeding management treatments in a fallow field: natural fallow, physical clearance, deep tillage, sprayed herbicide.

Weed species	Natural fallow	Physical clearance	Deep tillage	Sprayed herbicide
	N	C	D	H
Annual weed				
*Humulus scandens*	33.83	5.42	3.22	3.84
*Chenopodium serotinum*	17.97	12.22	12.82	18.52
*Potentilla supina*	8.67	5.49	15.94	12.91
*Descurainia sophia*	3.00	1.10	–	3.80
*Polygonum aviculare*	2.93	2.82	0.89	–
*Digitaria sanguinalis*	1.37	0.59	0.80	2.13
*Eleusine indica*	0.89	3.58	0.88	4.31
*Acalypha australis*	0.58	1.22	1.44	–
*Portulaca oleracea*	0.44	0.71	4.60	–
*Setaria viridis*	0.37	–	0.60	3.68
*Chenopodium album*	0.36	2.25	1.10	3.16
*Cyperus nipponicus*	–	1.14	–	1.05
*Cynodon dactylon*	–	0.50	0.26	1.90
*Amaranthus retroflexus*	–	–	1.00	–
*Euphorbia humifusa*	–	–	0.28	–
*Abutilon theophrasti*	–	–	0.28	–
*Hemistepta lyrata*	–	–	–	0.47
Biennial weed				
*Raphanus sativus*	3.55	0.92	–	2.48
*Lagopsis supina*	3.16	33.20	6.15	12.20
*Capsella bursapastoris*	1.20	5.88	7.62	1.16
Perennial weed				
*Calystegia hederacea*	15.16	15.47	32.62	15.63
*Belamcanda chinensis*	4.08	0.51	2.23	5.98
*Lilium concolor*	2.03	2.10	1.69	–
*Sonchus arvensis*	0.37	3.08	0.89	4.90
*Cephalanoplos setosum*	–	–	0.61	–
*Cirsium setosum*	–	0.64	3.94	0.44
*Ixeris polycephala*	–	1.15	0.12	1.42

**Note:**

*Raphanus sativus* and *Belamcanda chinensis* were in the fallow field before treatments and were regarded as “weeds”.

The RIV differed remarkably for different species of weeds. The total annual and biennial RIV in the C treatment was 75%, with the highest RIV (33%) being found in *Lagopsis supine*, a biennial weed. Against the C treatment, the RIV of perennial weeds in the D treatment was higher than those in other treatments, particularly for *Calystegia hederacea*, whose RIV was 33%, followed by *Chenopodium serotinum* and *Potentilla supine*. The dominant weed species in the N treatment were *Humulus scandens* and *Chenopodium serotinum*, with a total RIV of 52%. However, for the weeds in the H treatment, the distribution pattern was relatively uniform, with no species displaying an RIV more than 20%.

### Weed seed banks

Due to the difficulty in counting perennial weeds, the primary focus was on the weed seed banks of annual and biennial weeds. In total, we identified 12 annual and three biennial weed species in the soil weed seed bank (Table 2). *Potentilla supina* dominated the annual weed seed bank in different treatments, followed by *Portulaca oleracea, Chenopodium album, Chenopodium serotinum*, and others. No obvious difference was noted in weed seed banks between the pretreatment and posttreatment in the N treatments. However, *Potentilla supina*, *Chenopodium album*, *Cyperus nipponicus, Digitaria sanguinalis*, and *Capsella bursapastoris* all increased in the D treatment. In the D and H treatments, the weed seed banks were 83% and 24% higher, respectively, than those in the pretreatment. In contrast, the C treatment decreased the seed bank by 34% against the pretreatment, owing to the physical clearance (Table 2).

**Table 2:** Changes in annual and biennial weed seed banks under different weeding management treatments.

Weed species	Natural fallow				Physical clearance				Deep tillage				Sprayed herbicide			
	Pre-treatment		Post-treatment		Pre-treatment		Post-treatment		Pre-treatment		Post-treatment		Pre-treatment		Post-treatment	
	100 s m^−2^	%	100 s m^−2^	%	100 s m^−2^	%	100 s m^−2^	%	100 s m^−2^	%	100 s m^−2^	%	100 s m^−2^	%	100 s m^−2^	%
Annual weed																
*Potentilla supina*	58.00	55.47	53.78	55.76	55.56	56.95	35.00	54.03	82.67	73.30	137.22	66.43	61.89	53.46	75.11	52.28
*Portulaca oleracea*	13.56	12.96	11.78	12.21	7.33	7.52	10.22	15.78	12.22	10.84	14.78	7.15	11.83	10.22	19.67	13.69
*Chenopodium album*	13.33	12.75	9.44	9.79	4.33	4.44	6.22	9.61	2.89	2.56	10.22	4.95	8.11	7.01	12.44	8.66
*Chenopodium serotinum*	12.00	11.48	6.56	6.80	3.78	3.87	3.22	4.97	1.78	1.58	1.33	0.65	5.99	5.17	3.56	2.47
*Cyperus nipponicus*	1.33	1.28	1.44	1.50	3.33	3.42	5.67	8.75	5.56	4.93	14.44	6.99	14.56	12.57	3.78	2.63
*Acalypha australis*	0.56	0.53	0.10	0.10	0.44	0.46	–	–	0.56	0.49	–	–	–	–	0.89	0.62
*Hemistepta lyrata*	0.44	0.43	0.11	0.12	1.33	1.37	0.11	0.17	0.22	0.20	0.33	0.16	1.89	1.63	0.22	0.15
*Eleusine indica*	0.22	0.21	1.21	1.26	2.00	2.05	0.56	0.86	2.67	2.36	1.78	0.86	5.98	5.16	6.78	4.72
*Amaranthus retroflexus*	0.11	0.11	0.11	0.12	0.11	0.11	0.11	0.17	–	–	0.11	0.05	0.33	0.29	–	–
*Descurainia sophia*	0.11	0.11	1.20	1.24	2.33	2.39	0.56	0.86	–	–	–	–	0.83	0.71	5.67	3.94
*Setaria viridis*	0.11	0.11	2.33	2.42	–	–	0.22	0.34	0.67	0.59	–	–	–	–	0.44	0.31
*Digitaria sanguinalis*	–	–	4.27	4.42	4.00	4.10	1.44	2.23	1.11	0.99	16.67	8.07	0.33	0.29	1.56	1.08
Biennial weed																
*Capsella bursapastoris*	3.89	3.72	2.56	2.65	5.78	5.92	0.22	0.34	1.44	1.28	8.56	4.14	1.86	1.61	0.78	0.54
*Raphanus sativus*	0.67	0.64	1.11	1.15	2.56	2.62	0.56	0.86	0.44	0.39	0.67	0.32	1.11	0.96	0.78	0.54
*Lagopsis supina*	0.22	0.21	0.44	0.46	4.67	4.78	0.67	1.03	0.56	0.49	0.44	0.22	1.07	0.92	12.00	8.35
	104.56		96.44		97.56		64.78		112.78		206.56		115.77		143.67	

**Notes:**

*Raphanus sativus* was in the fallow field before treatments and were regarded as “weeds”.

100 s m^−2^, 100 weed seeds m^−2^; %, percentage contribution of weed species to the weed seed banks.

**Figure 1:**
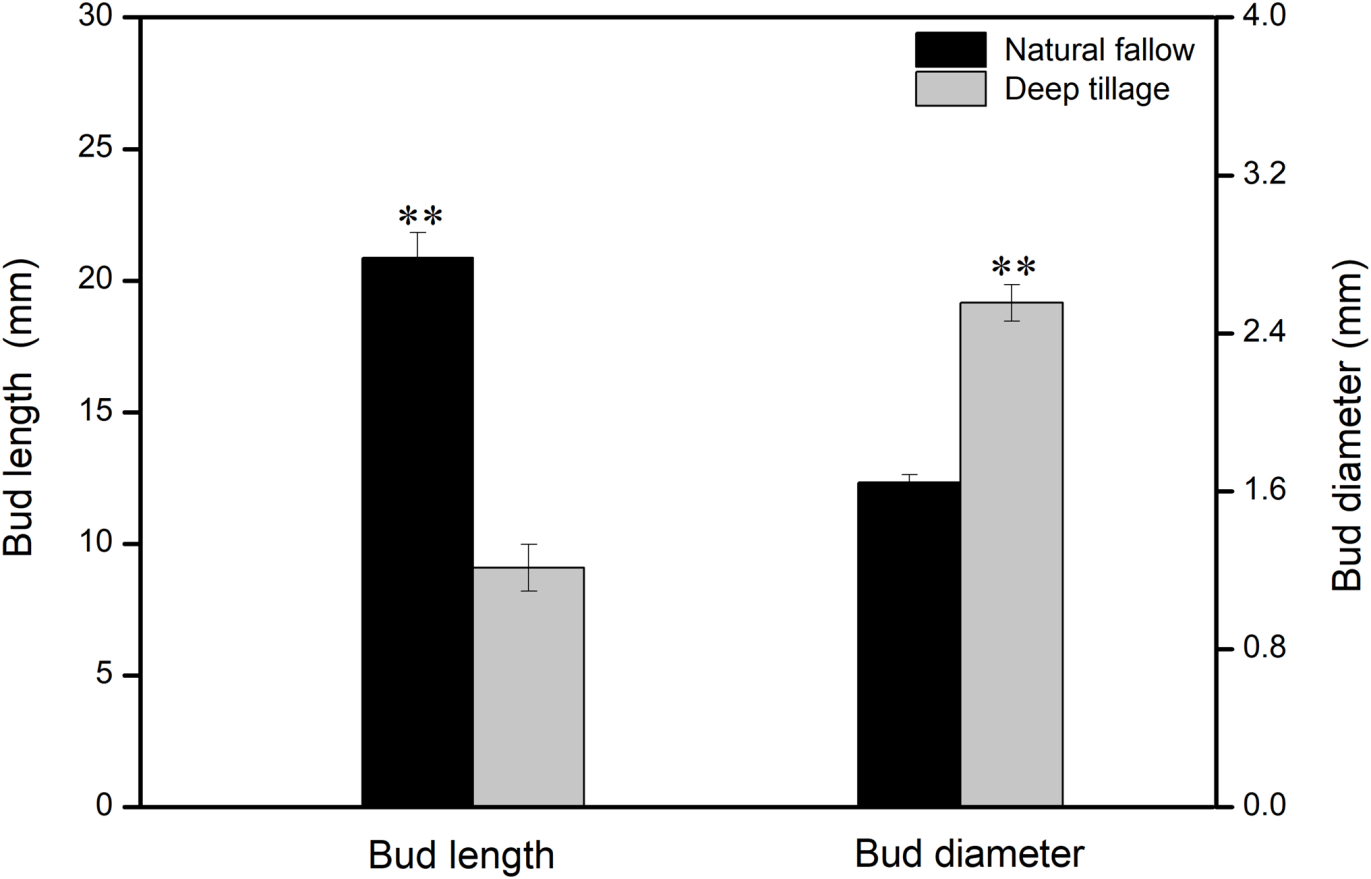
*Calystegia hederacea* rhizome bud length and diameter in soil layer (0–20 cm) with different weeding management treatments. Values are the mean ± standard error, *n* = 60. “**” shows a significant difference among treatments at *P* < 0.01.

The shoots and diameters of rhizomes of *Calystegia hederacea* were significantly affected by different treatments (Fig. 1). The average shoot length in the N treatment was 20.8 mm, more than twice that of deep tillage with a moldboard plow (D) (*P* < 0.01). The diameter of the rhizomes in the D treatment was significantly greater than that in the N treatment (*P* < 0.01).

### Soil nutrients

The total nitrogen content in the D treatment was significantly higher than that of other treatments in the 0–20 cm soil layer (*P* < 0.05, Table 3). However, the total phosphorus content in the D treatment was significantly lower than that of other treatments in 0–20 cm soil layer (*P* < 0.05). The lowest total nitrogen in the topsoil was noted in the C treatment, while the total phosphorus content in C was the highest in the 0–20 cm topsoil (0.93 g kg^–1^).

**Table 3:** Nutrients in the topsoil layers under different weeding managements in a fallow field: natural fallow, physical clearance, deep tillage, sprayed herbicide.

Soil nutrients	Topsoil (cm)	Natural fallow	Physical clearance	Deep tillage	Sprayed herbicide
Total nitrogen (g kg^−1^)	0–20	0.77 ± 0.01^b^	0.76 ± 0.01^b^	0.84 ± 0.03^a^	0.78 ± 0.00^b^
	20–40	0.54 ± 0.00^ab^	0.46 ± 0.01^c^	0.57 ± 0.02^a^	0.5 ± 0.02^bc^
Total phosphorus (g kg^−1^)	0–20	0.87 ± 0.02^a^	0.93 ± 0.02^a^	0.74 ± 0.04^b^	0.93 ± 0.03^a^
	20–40	0.56 ± 0.00^b^	0.68 ± 0.03^a^	0.6 ± 0.06^ab^	0.6 ± 0.01^ab^
Ammonium nitrogen (mg kg^−1^)	0–20	8.41 ± 0.41^a^	7.62 ± 0.40^a^	6.92 ± 0.78^a^	7.34 ± 0.67^a^
	20–40	8.69 ± 0.54^a^	5.99 ± 0.26^b^	6.06 ± 0.33^b^	6.68 ± 0.56^b^
Nitrate nitrogen (mg kg^−1^)	0–20	10.59 ± 0.61^b^	0.75 ± 0.02^b^	32.7 ± 7.76^a^	6.56 ± 0.95^b^
	20–40	7.03 ± 0.65^b^	0.91 ± 0.07^c^	10.6 ± 0.59^a^	6.36 ± 0.88^b^
Available phosphorus (mg kg^−1^)	0–20	23.34 ± 0.59^b^	22.8 ± 2.32^b^	29.52 ± 2.18^a^	25.73 ± 1.01^ab^
	20–40	11.57 ± 0.32^a^	15.82 ± 0.96^a^	15.88 ± 2.47^a^	13.68 ± 1.07^a^

**Note:**

The nutrients were compared among the treatments in the same topsoil layer, either 0–20 or 20–40 cm. Values were reported as the means ± standard error, *n* = 3. Within a row (topsoil layer), different lowercase letters indicate a significant difference among treatments (*P* < 0.05).

The available phosphorus content in the D treatment was the highest among the four treatments in the 0–20 cm soil layer (*P* < 0.05). The content of available nitrogen increased in the D treatment and was 108% higher than that in the N treatment. The content of nitrate nitrogen in the D treatment was also higher than those of other treatments for both soil layers, accounting for 83% of the available nitrogen. Nevertheless, the ammonium nitrogen content from all soil layers was much higher than that of the nitrate nitrogen component under C treatment.

The RIV of *Calystegia hederacea* displayed a significantly positive correlation with soil total nitrogen (*r* = 0.82, *P* < 0.01) and nitrate nitrogen (*r* = 0.87, *P* < 0.01) in the 0–20 cm soil layer (Table 4). However, a negative correlation was found between the RIV of *Calystegia hederacea* and the total phosphorus (*r* = −0.76, *P* < 0.01). Moreover, a significantly positive correlation was noted between the RIV of *Capsella bursapastoris* and the total nitrogen in 0–20 cm soils (*r* = 0.79, *P* < 0.01), as well as *Eleusine indica* (*r* = 0.61, *P* < 0.05). The RIV of *H. scandens* displayed a considerably positive correlation with ammonium nitrogen (*r* = 0.84, *P* < 0.01) in the 20–40 cm soil layer, which was different from other weeds.

**Table 4:** Correlation coefficient between the relative importance values of specific weeds and soil properties.

Soil property	Soil depth (cm)	*Humulus scandens*	*Lagopsis supina*	*Potentilla supina*	*Chenopodium serotinum*	*Capsella bursapastoris*	*Cirsium setosum*	*Eleusine indica*	*Calystegia hederacea*
Total nitrogen	0–20	−0.28	−0.30	−0.04	0.33	0.79**	0.48	0.61*	0.82**
	20–40	0.30	−0.50	−0.28	−0.16	0.54	0.14	0.25	0.60*
Total phosphorus	0–20	0.00	0.53	−0.08	−0.12	−0.55	−0.04	−0.01	−0.76**
	20–40	−0.49	0.43	−0.06	0.31	0.23	0.05	0.25	0.04
Ammonium nitrogen	0–20	0.49	−0.51	0.31	−0.23	−0.25	0.29	−0.02	−0.38
	20–40	0.84**	−0.19	−0.42	−0.50	−0.49	−0.42	−0.35	−0.39
Nitrate nitrogen	0–20	−0.09	−0.57	0.07	0.29	0.70*	0.34	0.41	0.87**
	20–40	0.13	−0.39	−0.09	−0.18	0.41	0.07	−0.01	0.65*
Available phosphorus	0–20	−0.33	−0.11	0.14	−0.04	0.46	−0.02	−0.32	0.58*
	20–40	−0.57	0.30	0.02	0.25	0.42	−0.02	0.07	0.32

**Notes:**

The weed community and soil samples were all surveyed on October 28, 2016. Eight most important specific weeds were chosen to perform this correlation analysis. The significant differences were compared in the same topsoil, namely, 0–20 and 20–40 cm.

* Significant differences at *P* < 0.05.

** Significant differences at *P* < 0.01.

### Soil microbial biomass

**Figure 2:**
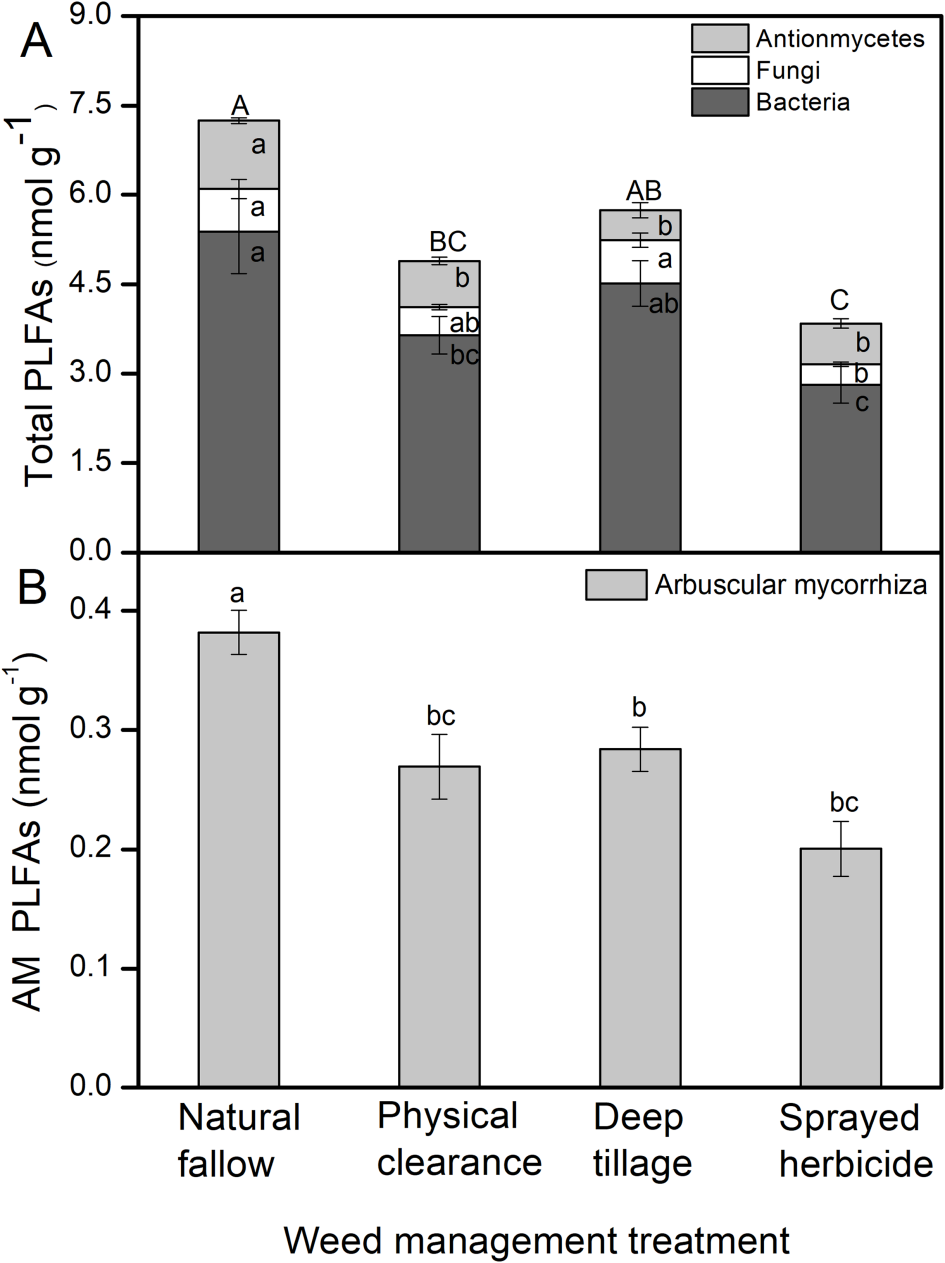
PLFAs of soil microorganisms under different weeding management treatments. Values are reported as the mean ± standard error, *n* = 3. (A) Total PLFAs with proportions for actinomycetes, fungi, and bacteria. Different capital letters show a significant difference at *P* < 0.05 for total PLFAs under the different treatments; different lowercase letters show a significant difference at *P* < 0.05 for actinomycetes, fungi, or bacteria under the different treatments. (B) Arbuscular mycorrhizal (AM) fungi PLFAs. Different lowercase letters show a significant difference at *P* < 0.05 under the different treatments.

The total PLFAs value was the highest in N treatment (7.2 nmol g^–1^), followed by D and C treatments. The total biomass of soil bacterial, fungal, and actinomycete in H treatment was the lowest among the four treatments, which was significantly less than N and D treatments (*P* < 0.05, Fig. 2A). Fungi biomass in the D treatment was the highest among all the treatments, and significantly greater than the H treatment (*P* < 0.05). AM fungal biomass was the highest in the N treatment (Fig. 2B) and lowest in the H treatment. The AM biomass in the N treatment exceeded those in C, D, and H treatments by more than 42%, 35%, and 91%, respectively.

**Figure 3:**
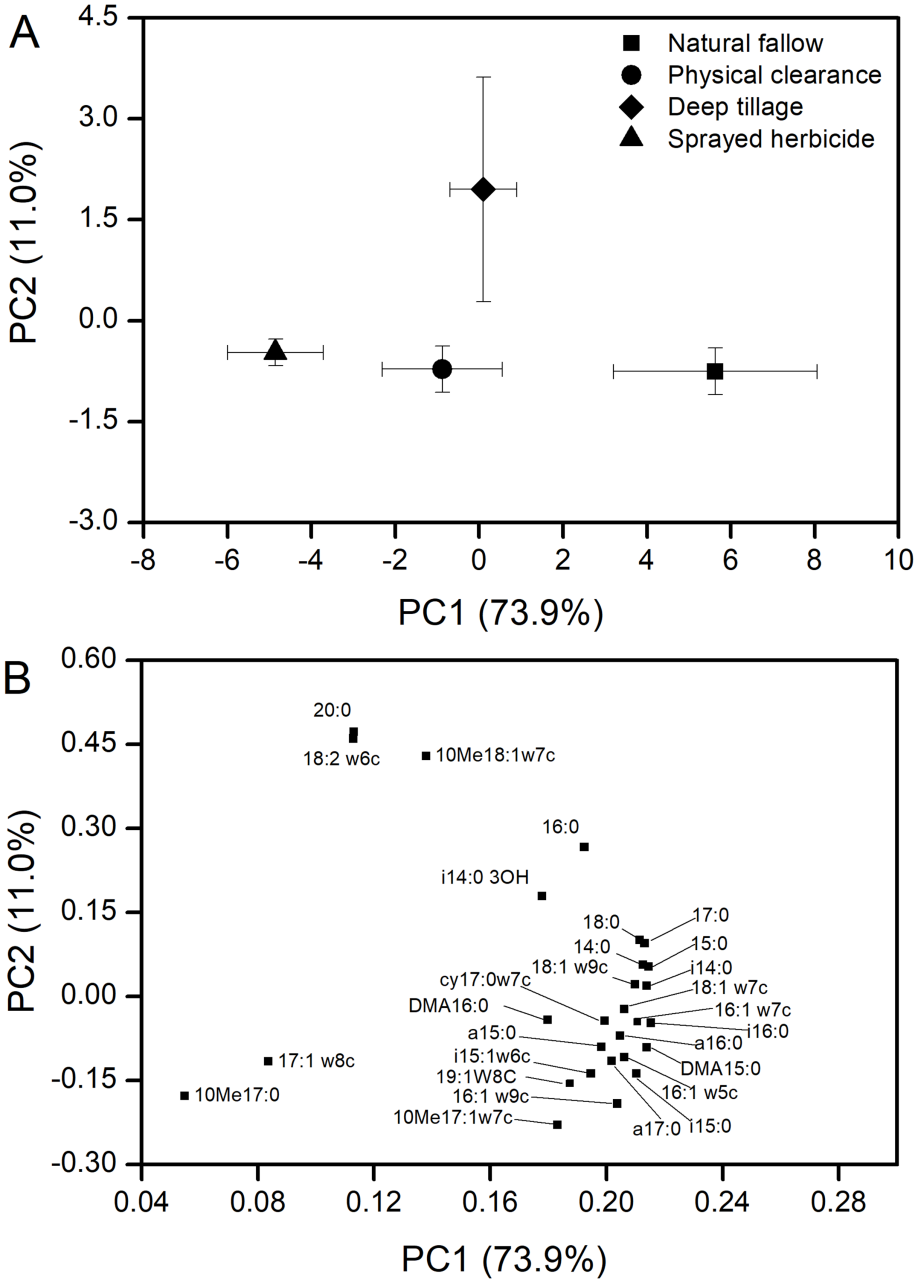
PCA of PLFAs data from soil samples (0–20 cm) at different weeding management treatments. Error bars indicated as ± standard error, *n* = 3. (A) PCA of soil microbial community structure within the two profiles. (B) Loadings of the individual PLFAs from the PCA of PLFAs data of principal components 1 and 2.

**Figure 4:**
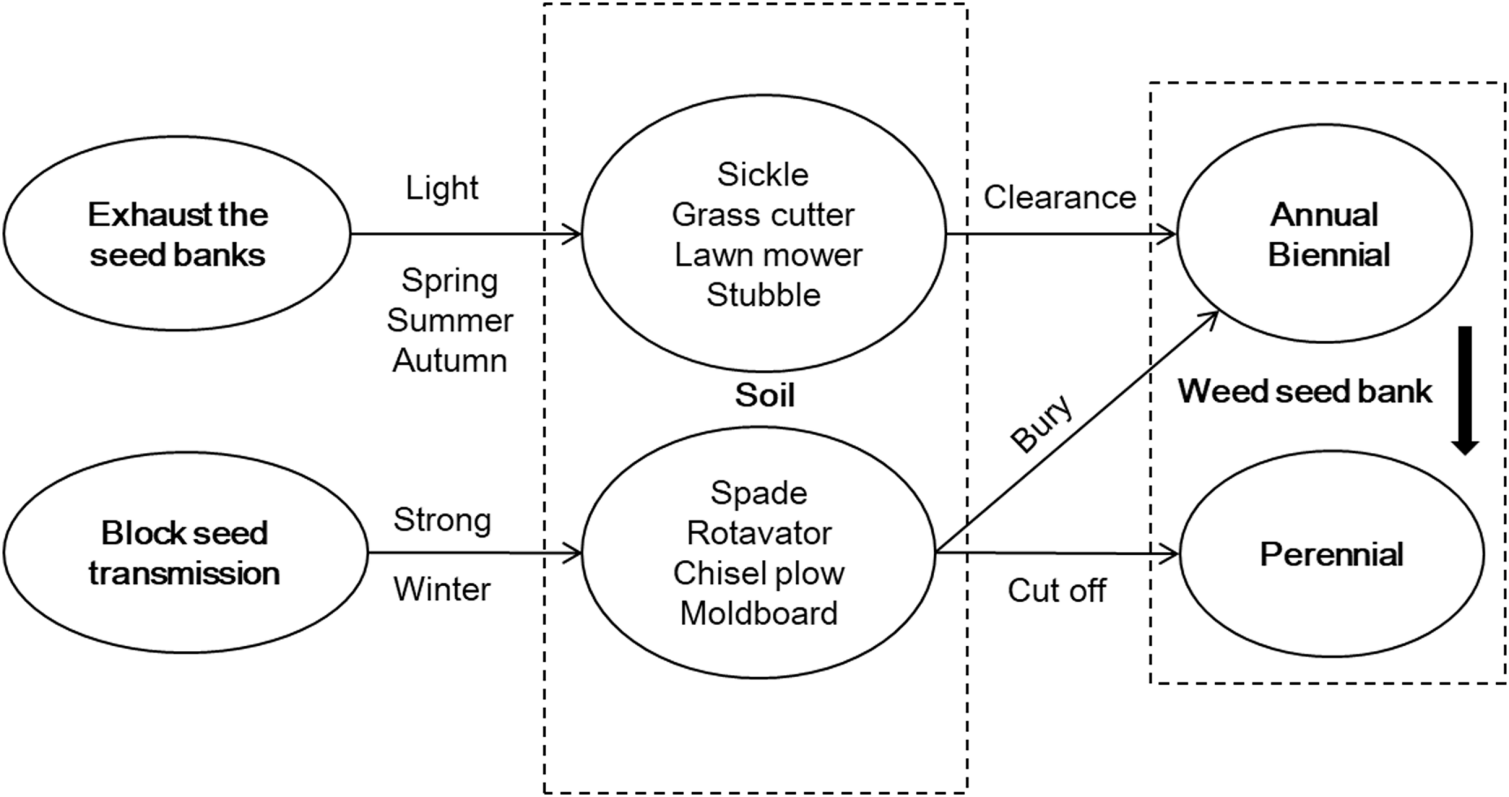
Strategy framework for weed management in a fallow field in Northern China. According to the characteristics of a weed seed bank, weed management strategy was proposed for a fallow field; namely, exhaust the seed banks, block seed transmission, and take advantage of natural opportunities to prevent weed growth.

Yielding 28 PLFAs were performed to PCA, with the first two principal components (PCs) accounting for 74% and 11% of the variance, respectively (Fig. 3A). Microbial communities from N and H treatment were clearly separated in the PC1, while the D treatment was isolated from the other three treatments in the PC2. The PC loadings revealed that almost all PLFAs were positively correlated with PC1, e.g., 15:0DMA, 15:0, i14:0, i16:0, 16:1w7c, and 18:1w7c (Fig. 4B). The dominant PLFAs, e.g., 18:2w6c, 10Me18:1w7c, and 20:0 were positively correlated with PC2. Conversely, the 16:1w9c, 10Me17:0, 10Me17:1w7c, and so on were negatively correlated with PC2.

## Discussion

### Different weed managements affected the weed seed bank

The mature seeds of weeds are the primary contributors to a weed seed bank. Here, the weed seed distribution pattern in a naturally fallowed land was similar to that under no-tillage conditions, with most of the seeds being primarily distributing in the soil surface (Nichols et al., 2015). Such a distribution pattern might maintain a high level of weed germination. Thus the composition of the weed seed bank could be affected by different weeding treatments. For instance, in the N treatment, *H. scandens* became a dominant species (Table 1) because of its sprawl and spatially competitive ability. These characteristics could also prevent other weeds from producing huge seeds. The effect of herbicide on the germinated weeds was as expected, however, herbicide application played a limited role in controlling the capacity of the soil weed seed bank (Table 2). This may be due to the fact that once weeds grow vigorously again, the seed bank capacity recovers quickly. Physical clearance (C) consumed most of the weed seed bank, particularly for annual and biennial weeds, e.g., *Potentilla supina* and *L. supinais*, *Digitaria sanguinalis* (Table 2). The annual and biennial weed seeds increased after D treatment, primarily because deep plowing buried some weed seeds in the topsoil (Mohler & Galford, 2008). In the D treatment, the RIV of *Calystegia hederacea* was higher because rhizomes continued to reproduce before entering the dormant period in autumn and winter (Ito, Takagi & Yoshino, 2005). Two primary types of rhizomes were observed after deep tillage: rhizomes with or without nodes. For rhizomes with nodes, new plants germinated and grew, in contrast to those without nodes. In this experiment, the length of shoots with reproductive rhizomes after deep tillage in winter was remarkably inhibited (Fig. 1). The decrease in reproductive rhizomes occurred because deep plowing cut off the rhizomes of the perennial weeds from the bottom and brought them to the surface, where they died of exposure or frost (Qiang, 2009).

According to our findings, we considered that the combination “C + D” might be the suitable approach to reduce weed seed bank in a fallow land. Specifically, we could employ the “C” category before weeds blossom and seed, and promote the germination of annual and biennial weed seeds under suitable phenological conditions, such as in the spring, summer, and autumn seasons. The “D” category could be applied in winter before the topsoil freezes, which may help prevent the underground propagation of perennial weeds and also bury some annual and biennial weed seeds. However, we need a further research to test whether the “C + D” category could maximize the consumption of different life form seed banks.

### Effects of different weed management practices on soil quality of fallow land

The different weeding treatments also influenced the soil quality. The available nitrogen and available phosphorus content was higher in the D treatment than in other treatments (Table 3). Those elements likely came from decomposed weeds (Zhao, 1986). However, the total P concentration was lower in the D treatment, which might be due to the fact that P leaching happened in the rainfall season (Xue et al., 2013). The C treatment increased the coverage of bare soil, which possibly caused leaching of the soil available nitrogen. According to a global meta-analysis, more than 60% of soil nitrogen was lost from a bare field vs a fallow field (Wortman, 2016). Most seriously, in the Liaoning Province, China, researchers found that nitrogen released from weeds reached to 60.3% after a burial period of only 3 months (Zhao, 1986). In this study, the D treatment increased the proportion of nitrate nitrogen, which was consistent with the findings of previous investigators (Nyborg & Malhi, 1989; Wang et al., 2015). The incorporation of arable plant residues may delay the leaching of nitrate nitrogen, which could be a reasonable solution to improve soil nitrogen reservoirs (Mitchell et al., 2000). The soil nutrients may also affect the emergence of weeds (Table 3), however this effect might be faint due to the short period of seed generation.

Different weed management practices could disturb the soil microbial biomass and community structures. The total PLFAs was the highest in the N treatment, which could be due to the effects from plant litter (Wang et al., 2011). The total PLFAs and AM fungi were the lowest in H treatment among all the four treatments (Fig. 2). There are advantages to herbicide use in conventional agriculture, providing savings in time, labor, and costs compared with artificial weeding. However, herbicides cause serious pollution to the environment and food, which may in turn affect human health (Annett, Habibi & Hontela, 2014; Gomiero, Pimentel & Paoletti, 2011; Mesnage et al., 2017). AM fungi plays an important role in plant nutrient utilization and disease reduction (Johansson, Paul & Finlay, 2004). Thus, the degradation of AM fungi can directly reduce the quality of cultivated land and affect the nutrient utilization in the follow-up crop. Herbicides are able to affect AM fungi when a second crop is planted through their effects on plant metabolism and growth (Makarian et al., 2016). Soil microbial communities and soil respiration increased susceptibility to reductions in soil pH from acidity to neutrality (5.5–7.5) with the application of glyphosate (Nguyen et al., 2016), which was consistent with the results of the current study. A higher content of available phosphorus was noted in the H treatment, which might be associated with the phosphorus supplied by the mineralization of glyphosate (Forlani et al., 1999).

### Weed management in a fallow field

The weeds in a fallow field are not only the “seed stock” but also the “seed source.” Weed species are typically “r-strategists,” with characteristics that include a short life expectancy, high seed productivity, and rapid growth. The primary purpose of weed management is to control and reduce the weed seed bank in the soil (Gallandt, 2014). Weeding at the correct time can relieve the possibility of an eruption of seed rain and weaken the propagation of seeds, which is believed to be an effective means to prevent weeds from rebuilding their populations (Nichols et al., 2015). Weeding methods are numerous and include artificial weeding, mechanical weeding, crop rotation, straw mulching, etc. However, artificial weeding is labor-intensive and is not suitable for large areas. Therefore, mechanical weeding might be an alternative approach to minimize labor input, though improper mechanical weeding can destroy the soil structure and fertility (Smith et al., 2011).

Based on the weed characteristics found in this research, a weed management strategy was proposed for a fallow field, namely, exhausting seed banks, blocking seed transmission, and taking advantage of natural opportunities to prevent weed growth (Fig. 4). To exhaust the seed banks, the topsoil was lightly disturbed to break the dominant annual and biennial weed seeds and to promote weed community succession. This was followed by clearing the seedlings by machine. To block seed transmission, a strong disturbance with deep tillage was applied to the soil in winter for the perennial weeds, such as *Calystegia hederacea*, which exposed the rhizomes to the open air, where they died of low temperatures and frost. Meanwhile, deep tillage could also bury some annual and biennial weed seeds to prevent germination. Weeding was required before seed rains began in order to take advantage of the natural opportunity to prevent weed growth.

## Conclusions

Herbicides had a limited inhibitory effect on weed seed banks during the fallow period of the farmland but reduced the total amount of soil microorganisms. The timely removal of surface weeds mostly reduced the annual and biennial weed seed banks, whereas deep tillage improved the soil physical and chemical properties and interfered with rhizome propagation of perennial weeds during winter. A weed control strategy was proposed in fallow land through approaches such as exhausting seed banks, blocking seed transmission, and taking advantage of natural opportunities to prevent weed growth. The flexible use of weeding methods can not only reduce the degree of weed damage but also improve the soil quality. Our findings might provide a theoretical basis for weed management in fallow lands or in organic farming systems.

## Supplemental Information

10.7717/peerj.7650/supp-1 Supplemental Information 1 Raw data for Fig. 1.

10.7717/peerj.7650/supp-2 Supplemental Information 2 Raw data for Fig. 2.

10.7717/peerj.7650/supp-3 Supplemental Information 3 Raw data for Fig. 3.

10.7717/peerj.7650/supp-4 Supplemental Information 4 Relative importance values (%RIV) for all types of weeds under the different weed management treatments in a fallow field: natural fallow, physical clearance, deep tillage, sprayed herbicide.

10.7717/peerj.7650/supp-5 Supplemental Information 5 Raw data for Table 3.

10.7717/peerj.7650/supp-6 Supplemental Information 6 Raw data for Table 4.

## Additional Information and Declarations

### Field Study Permissions

The following information was supplied relating to field study approvals (i.e., approving body and any reference numbers): The field experiment was approved by the Plant Eco-physiological Research Group in the Institute of Botany, Chinese Academy of Sciences (201601.8).

### Data Availability

The following information was supplied regarding data availability: The raw measurements are available in the Supplemental Files.
